# The Impact of a Natural Olive-Derived Phytocomplex (OliPhenolia^®^) on Exercise-Induced Oxidative Stress in Healthy Adults

**DOI:** 10.3390/nu14235156

**Published:** 2022-12-04

**Authors:** Justin D. Roberts, Joseph Lillis, Jorge Marques Pinto, Ashley G. B. Willmott, Lata Gautam, Christopher Davies, Álvaro López-Samanes, Juan Del Coso, Havovi Chichger

**Affiliations:** 1 Cambridge Centre for Sport and Exercise Sciences, School of Psychology and Sport Science, Anglia Ruskin University, Cambridge CB1 1PT, UK; 2 School of Life Sciences, Anglia Ruskin University, Cambridge CB1 1PT, UK; 3 Exercise Physiology Group, Faculty of Health Sciences, Universidad Francisco de Vitoria, 28223 Madrid, Spain; 4 Centre for Sport Studies, Rey Juan Carlos University, 28943 Fuenlabrada, Spain

**Keywords:** polyphenols, OliPhenolia^®^, oxidative stress, exercise, nutrition, antioxidants

## Abstract

The role of natural polyphenols in reducing oxidative stress and/or supporting antioxidant mechanisms, particularly relating to exercise, is of high interest. The aim of this study was to investigate OliPhenolia^®^ (OliP), a biodynamic and organic olive fruit water phytocomplex, rich in hydroxytyrosol (HT), for the first time within an exercise domain. HT bioavailability from OliP was assessed in fifteen healthy volunteers in a randomized, double-blind, placebo controlled cross-over design (age: 30 ± 2 yrs; body mass: 76.7 ± 3.9 kg; height: 1.77 ± 0.02 m), followed by a separate randomized, double-blinded, cohort trial investigating the short-term impact of OliP consumption (2 × 28 mL∙d^−1^ of OliP or placebo (PL) for 16-days) on markers of oxidative stress in twenty-nine recreationally active participants (42 ± 2 yrs; 71.1 ± 2.1 kg; 1.76 ± 0.02 m). In response to a single 28 mL OliP bolus, plasma HT peaked at 1 h (38.31 ± 4.76 ng∙mL^−1^), remaining significantly elevated (*p* < 0.001) until 4 h. Plasma malondialdehyde (MDA), superoxide dismutase (SOD), catalase (CAT), reduced glutathione (GSH) and HT were assessed at rest and immediately following exercise (50 min at ~75% $\dot{V}$O_2_max then 10 min intermittent efforts) and at 1 and 24 h post-exercise, before and after the 16-day supplementation protocol. Plasma HT under resting conditions was not detected pre-intervention, but increased to 6.3 ± 1.6 ng·mL^−1^ following OliP only (*p* < 0.001). OliP demonstrated modest antioxidant effects based on reduced SOD activity post-exercise (*p* = 0.016) and at 24 h (*p* ≤ 0.046), and increased GSH immediately post-exercise (*p* = 0.009) compared with PL. No differences were reported for MDA and CAT activity in response to the exercise protocol between conditions. The phenolic compounds within OliP, including HT, may have specific antioxidant benefits supporting acute exercise recovery. Further research is warranted to explore the impact of OliP following longer-term exercise training, and clinical domains pertinent to reduced oxidative stress.

## 1. Introduction

Strenuous exercise requires an increased oxygen consumption, creating a disturbance in the intracellular pro-oxidant–antioxidant homeostatic axis [1]. The impaired balance between free radical production and antioxidant pathways [2] results in net oxidative stress. Such oxidative stress is due to either the overproduction of reactive oxygen and nitrogen species (RONS), including the superoxide anion (O_2_^•−^), hydrogen peroxide (H_2_O_2_), hydroxyl (^•^OH), peroxyl (ROO^•^) and peroxynitrate (ONOO^−^) radicals [3], or due to an insufficient endogenous antioxidant defense system, including both enzymatic and non-enzymatic antioxidants [1]. To an extent, however, production of RONS associated with exercise may serve of benefit to produce long-term physical and physiological adaptations induced by exercise training. This may occur via the stimulation of stress signaling pathways, increasing the expression of genes encoding cytoprotective proteins such as nuclear factor erythroid 2-related factor 2 (*Nrf2*) [4,5], the activation of sirtuins (*SIRT1*) and increased activity of peroxisome proliferator activated receptor γ coactivator (*PGC-1α*) [3]. However, over-disturbance or sustained imbalance between RONS production and antioxidant pathways could disrupt acute recovery, metabolic and signaling mechanisms in a negative manner [6], potentially supporting the need for exogenous antioxidant nutrients.

Polyphenols are the most abundant antioxidant in the diet, with over 8000 polyphenolic compounds identified in a range of plant species [7]. As widespread constituents of fruits, vegetables, cereals, chocolate, and beverages such as coffee, tea and wine [8], an average 100 g fresh weight of fruits (grapes, apples, pears, cherries, and berries) contains up to 300 mg of polyphenols [9]. Primarily occurring in conjugated forms, polyphenols are secondary plant metabolites, with highly diverse chemical structures [10], typically characterized by two or more hydroxyl groups attached to one or more benzene rings [11]. As natural compounds, polyphenol nutrients possess multiple biological activities [12,13], and an expanding body of evidence has demonstrated anti-inflammatory [14], immunomodulatory [15] and antioxidant responses [1] to various polyphenol interventions. As such, there is current interest in the application of polyphenol nutrients as adjunct dietary strategies to support exercise training or sports performance in terms of promoting antioxidant mechanisms or attenuating exercise-induced muscle damage following acute strenuous bouts [16].

The mechanisms responsible for enhanced antioxidant capacity from polyphenol consumption have been attributed to the suppression of reactive oxygen species (ROS) formation, scavenging of ROS and the upregulation of antioxidant defenses [17,18], ultimately exerting anti-inflammatory responses due to perturbations in homeostasis [19]. Depending on the exercise setting, this could translate to accelerating post-exercise recovery time, lowering oxidative stress (and endogenous enzyme requirements) and reducing exercise-induced muscle damage. Certain polyphenol compounds act as in vivo antioxidants and enhance the expression of genes encoding enzymes such as superoxide dismutase (SOD), catalase (CAT) and glutathione peroxidase (GPx), thereby supporting endogenous antioxidant levels [20]. This ultimately compliments the maintenance of redox balance within the body [21] which combined with the synergistic approach of exogenous antioxidants further support homeostatic processes. Similar pro-homeostatic results have been demonstrated when exogenous antioxidants are consumed alongside exercise training. A recent review [22] highlighted that the addition of polyphenol-rich, food-based products in days surrounding strenuous or damaging exercise accelerated the recovery of muscle function by up to 13% and reduced muscle soreness by up to 29%. Additionally, consumption of polyphenol supplementation has been shown to reduce markers of oxidative stress and inflammation [23,24,25] and support acute exercise recovery kinetics [26]. This could hold potential interest in supporting adaptations to physical exercise.

There is current interest in olive-derived polyphenols, in particular the benefits of hydroxytyrosol (HT), oleuropein and verbascoside in supporting antioxidant mechanisms [27]. For example, HT has been evidenced to positively upregulate antioxidant defenses [28] as well as effectively modify oxidative stress markers [29]. However, there is a paucity of research supporting the potential use of HT to modulate exercise-induced oxidative stress. Commercially available OliPhenolia^®^ (OliP) is a polyphenol and HT-rich phytocomplex produced through a process of concentration, reverse osmosis and mechanical filtering of the aqueous part of olives. The resulting concentrated olive fruit water is currently being explored for its clinical and health applications (in addition to environmental sustainability). This is the first study to investigate the impact of OliP within an exercise domain. This project involved two aims: (i) to investigate the bioavailability of HT as the main compound within commercially available OliP in healthy, adult volunteers; and (ii) to investigate the short-term use of OliP on markers of oxidative stress in response to acute aerobic exercise in recreationally active, healthy volunteers compared to a placebo.

## 2. Materials and Methods

### 2.1. Ethical Approval and Trial Registration

This research employed a two-phase approach encompassing a bioavailability study and separate main exercise study. For both phases, institutional ethical approval was obtained from the Faculty of Science and Engineering Research Ethics Panel, Anglia Ruskin University (Ethical approval number: FSE/FREP/20/946) and was conducted in accordance with the Declaration of Helsinki (2013). The study was also registered with clinicaltrials.gov (ID: NCT04959006).

### 2.2. Phase 1—Bioavailability Study

#### 2.2.1. Study Participants and Eligibility

As part of an initial study, and following a priori power calculation (G*power3, Dusseldorf, Germany [30]); using α = 0.05; 1 − β = 0.80, based on observed plasma HT responses compared with controls [31], a minimum sample size of 6 was estimated. To meet study eligibility, participants were required to complete a study briefing and health screen questionnaire, provide written, informed consent, and be >21 yrs. Participants with a known history of cardiovascular, metabolic or blood-related disorders were not eligible for study inclusion. This extended to individuals with recent viral infections including COVID-19, or those taking any prescribed medication or consuming nutritional supplements. Additionally, participants with a known intolerance or allergy to olives or prunes were excluded. Seventeen healthy participants volunteered for the bioavailability phase and registered as ‘recreationally active’ (defined as general exercise activity 1–3 times per week). Two participants withdrew prior to the start of the study due to personal reasons. Fifteen participants therefore took part (8 females, 7 males; mean ± standard error [S.E.], age: 30 ± 2 yrs; body mass: 76.7 ± 3.9 kg; height: 1.77 ± 0.02 m; body mass index: 24.8 ± 1.3 kg·m^2^).

#### 2.2.2. Bioavailability Trial Design and Procedures

This initial study employed a randomized, double-blinded, placebo-controlled crossover design. Participants attended the Human Physiology Laboratory, Cambridge Centre for Sport and Exercise Sciences, Anglia Ruskin University on two occasions separated by at least 7 days. Following a standardized overnight fasting period, upon arrival participants were assessed for body mass (Seca 780, Hamburg, Germany) and height (Seca CE123, Hamburg, Germany) before resting in a comfortable recumbent position. An intravenous 20-gauge cannula (Terumo Versatus™, Terumo Europe, Leuven, Belgium) was inserted by a qualified phlebotomist into a suitable forearm vein prior to sample collection at rest and at 60, 120, 240 and 480 min following consumption of one of the nutritional products outlined below (see Section 2.2.3). After each collection, samples lines were flushed with a 5 mL 0.9% sodium chloride solution. Venous whole-blood was collected into 4 mL Vacuette™ K2EDTA tubes (Greiner Bio-One GmbH, Kremsmunster, Austria), immediately centrifuged at 2000 rcf for 10-min, with plasma then extracted into sterile polypropylene cryovials (Fisherbrand, Fisher Scientific, Loughborough, UK) and frozen at −80 °C for later assessment of HT concentration (see Section 2.2.4).

#### 2.2.3. Nutritional Products

Following initial resting blood sample collection, participants were independently provided with a small sealed jar in a double-blinded manner, containing either: (i) 28 mL OliP (Fattoria La Vialla, Castiglion Fibocchi, Arezzo, Italy) or (ii) 28 mL colour and taste matched placebo (PL) containing equal parts prune juice (Sunsweet California Prune Juice, Yuba City, CA, USA), diet cola (Tesco Cola, Tesco, UK) and tonic water (Tesco low calorie Indian tonic water, Tesco, UK). Participants were requested to consume the jar immediately, after which the time for the bioavailability trial was started. The duration of the bioavailability trial was based on the company stipulation to consume 2 jars per day, each jar consumption separated by approximately 6 h. This phase therefore aimed to assess the acute bioavailability of HT from a single serve.

The OliP phytocomplex was produced from biodynamic, organic olives and supplied by Fattoria La Vialla independently of the main study. The sweetened version of the product contained 11.25 g olive extract, 12.12 g concentrated grape juice and 1.62 g lemon juice per jar. As part of preparations, pilot work was undertaken across several boxes of filled and empty jars to determine a mean filled weight of 81.1 ± 0.9 g per jar, with an estimated mean contents volume of 28.0 ± 1.0 mL per jar. Based on extensive pilot work, the devised PL drink provided a color, appearance and taste match and was therefore utilized across this research. All products were maintained under refrigerated conditions prior to use to maintain freshness.

Independent laboratory analysis was undertaken on samples of both products (Analytical Group SRL, Florence, Italy) using an internal method based on the determination of biophenols according to the International Olive Council method [32] using liquid chromatography with a detector wavelength of 280 nm (with results expressed in g of tyrosol·L^−1^ water using an internal standard). Overall findings are shown in Table 1 and converted to provide estimated analyses per 28 mL serve.

#### 2.2.4. Hydroxytyrosol (HT) Analysis

Gas Chromatography-Mass Spectrometry (GC-MS) analysis was performed with an Agilent 7820A GC (Santa Clara, CA, USA) interfaced with a 5977B MSD fitted with a ZB1 column (30 m × 0.25 mm × 0.25 μm). Injection volume was 2 μL at 280 °C in splitless mode, with helium carrier gas. The oven temperature was programmed to 80 °C and ramped to 200 °C at 15 °C·min^−1^, then ramped to 280 °C at 25 °C·min^−1^ and held for 3-min. The MSD transfer line was set to 250 °C, and source 230 °C. To remove sample carry over, ethyl acetate blanks were run between each sample.

For HT analysis, GC-MS instrument response was initially calibrated, and a calibration curve was produced by running pure standards of commercially available HT in triplicate at concentrations between 0.025 to 2 μg·mL^−1^ in full scan mode (70–400 *m*/*z*). *M*/*Z* ion ratios were identified for both HT and the internal standard (3-(4-hydroxyphenyl)-1-propanol). For sample preparation, 250 µL of plasma was prepared in the presence of an internal standard. The individual plasma samples were extracted using a liquid-liquid extraction method following acidic hydrolysis [33]. The solvent was evaporated from the extract and derivatized with 50 µL of N,O-bis-(trimethylsilyl)trifluoroacetamide + 1% trimethylchlorosilane (BSTFA + 1% TMCS) and 50 µL ethyl acetate.

Analysis was performed with GC-MS in selective ion monitoring mode (SIM). For detection and confirmation of HT and the internal standard, relative retention factors, three mass ions, and mass ion ratios were identified. Quantifying ions were *m*/*z* 267 and 206 for hydroxytyrosol and the internal standard respectively, and the corresponding qualifier ions were *m*/*z* 179, 193, and 370 for hydroxytyrosol, and *m*/*z* 191 and 296 for the internal standard. For quantification, peak area ratios (HT/internal standard) were converted to HT concentrations by comparison to the previously obtained HT GC-MS calibration curve. Data analysis was performed using Agilent MassHunter Workstation Software (vB.07.00, Santa Clara, CA, USA).

### 2.3. Phase 2—Main Intervention Study

#### 2.3.1. Study Participants and Eligibility

Initial power calculations using α = 0.05 and 1 − β = 0.80 estimated a group sample size of 13 based on previous research [34] involving a similar approach to the current study. Data for changes in SOD, GPx and CAT activity were used, with the highest estimate taken for a priori assessment. Participants were invited to volunteer based on the same inclusion criteria as the bioavailability phase. Additionally, however, participants were required to be free from any musculo-skeletal injury or any other reason that would prevent participation in vigorous cardiovascular exercise. Furthermore, participants were required to be deemed ‘recreationally active’ based on general exercise patterns 1–3 times per week and have a baseline maximal oxygen uptake ($\dot{V}$O_2max_) of >25.0 mL·kg^−1^·min^−1^ assessed at the preliminary laboratory visit. All participants provided written, informed consent prior to study inclusion.

From an initial pool, 32 healthy adults volunteered to take part in the study. Prior to final analysis, 2 participants were withdrawn for not completing the laboratory exercise task required, and 1 participant was withdrawn for not adhering to the general specifications of the study. Therefore, 29 participants completed the study. Baseline participant characteristics are shown in Table 2, including group distribution (see Section 2.3.2).

#### 2.3.2. Study Design, Laboratory Procedures and Nutritional Intervention

For the main intervention study, a randomized, double-blinded, placebo-controlled cohort design was implemented. Participants attended the Performance Testing Laboratory, Cambridge Centre for Sport and Exercise Sciences, Anglia Ruskin University under controlled environmental conditions (temperature: 19.6 ± 0.3 °C; barometric pressure: 1005.6 ± 1.2 mBar; and relative humidity: 48.4 ± 2.2%) on five occasions in an overnight fasted and euhydrated state as outlined below.

*Initial familiarization trial:* prior to the main intervention, all participants underwent an initial screening and familiarization session. Following a 10 min rest period, height (Seca CE123, Hamburg, Germany), body composition (Tanita SC-330ST, Amsterdam, The Netherlands) and blood pressure (Omron 705CP, Kyoto, Japan) were assessed. Participants then completed a two-part graded exercise treadmill test (GXT, using an h/p/cosmos Quasar Med treadmill, Nussdorf, Germany) in a similar manner reported elsewhere [35]. For GXT1, following a standardized 5 min warm-up, participants completed incremental exercise (increasing by 1.0 km·h^−1^ each 4 min with gradient fixed at 1%) until blood lactate exceeded 4.0 mmol·L^−1^ (Biosen C_Line, EKF Diagnostics, Cardiff, UK).

After a 10 min passive recovery period, participants then completed GXT2 starting at a speed 2.0 km·h^−1^ below the previous GXT1 final speed, with gradient increasing by 1% each minute until volitional exhaustion. Throughout both GXT protocols, continuous breath-to-breath respiratory assessment (MetaLyzer 3B, Cortex Biophysik, Leipzig, Germany) and telemetric heart rate monitoring (Polar Electro Ltd., Kempele, Finland) were captured. $\dot{V}$O_2max_ was estimated using the highest 15 breath rolling average oxygen consumption, with maximal individual effort determined through previously referenced criteria [36].

*Pre-intervention trial:* The pre-intervention trial took place over a 2 consecutive day period. Upon arrival on day 1, participants were rested in a recumbent position for 10 min prior to assessment of resting blood pressure and heart rate (Omron 705CP, Kyoto, Japan); after which venous whole blood was collected by a qualified phlebotomist into triplicate 4 mL Vacuette™ K2EDTA tubes (Greiner Bio-One GmbH, Kremsmunster, Austria), and immediately centrifuged, plasma extracted and stored in a similar manner previously described (see Section 2.2.2) for subsequent biochemical analysis (see Section 2.3.4). Anthropometric measures were then undertaken in a similar manner to the familiarization trial.

After a 5 min warm-up period, based on individual lactate and $\dot{V}$O_2max_ data from the previous GXT, participants then undertook a 60 min aerobic running session on the same treadmill, with the first 50 min based on steady-state exercise at 60% of the difference between workloads corresponding to both lactate threshold (LT) and lactate turnpoint (LTp). This ∆60% LT-LTp corresponded with an individual workload of ~75% $\dot{V}$O_2max_ and was based on previous research involving moderate domain exercise on oxidative stress biomarkers [37,38,39]. For the remaining 10 min of this session, participants performed 1 min intervals at an individual intensity 10% above LTp, interspersed with 1 min bouts at ∆60% LT-LTp.

Upon completion, participants were rested in a recumbent position with further venous blood samples collected immediately and 1 h post exercise, returning the next morning (overnight fasted) for a 24 h post exercise sample collection by the same phlebotomist. Having completed the 24 h resting blood sample, participants then performed GXT1 and GXT2 in the same manner previously described, with GXT2 serving as a means to assess time to exhaustion with verbal encouragement standardized for all participants.

*Nutritional intervention:* Participants were randomly assigned to either PL or experimental (OliP) treatment using a random number generator (Research Randomizer, www.randomizer.org (accessed on 10 May 2021)) and provided with a 16-day supply, with instructions to consume 2 jars per day (~56 mL total) between meals separated by ~6 h. This dosage and timeframe were based on company recommendations and product supply. Nutritional supplements were identical to those used in the bioavailability trial (see Section 2.2.3), with OliP supplied independently by Fattoria La Vialla (batch no.14, Castiglion Fibocchi, Arezzo, Italy). As a means to further blind conditions, PL supplements were provided in the same boxed packaging and jars as OliP and supplied in a double-blinded manner. To minimize likelihood of dietary intake influence, participants were also supplied with a list of common polyphenol-rich foods and requested to a consume a low polyphenol diet in the 72 h prior to each lab visit.

*Post-intervention trial:* Participants completed a post-intervention trial over a 2-day consecutive period in the same manner as the pre-intervention trial described above, with venous blood samples undertaken at rest, immediately post and at 1 h and 24 h after the initial aerobic running session. The first day of the post-intervention trial coincided with the last day of allocated nutritional supplements. On the final day of testing, participants were required to return all consumed jars, with any non-consumed taken into consideration as part of adherence monitoring (see Section 2.3.3).

#### 2.3.3. Exercise Activity, Dietary Monitoring and Supplement Adherence

Exercise activity and dietary monitoring were undertaken in a similar manner to that previously reported by our group [36,40]. Prior to, and across the full 16-day intervention, participants completed a standardized daily activity log to account for habitual exercise patterns based on session type, mean session heart rate, exercise duration and session perceived exertion (sRPE). Participants were requested to maintain typical exercise patterns, and be consistent across each 8-day period of the 16-day intervention. Training diaries were assessed by the same researcher for comparison of training load (exercise duration × sRPE), monotony (reflective of the training variability across days) and strain (training load × monotony to reflect overall accumulated exercise stress on the individual; [41]) to ensure relative consistency between intervention cohorts (Table 3).

For habitual dietary consistency, participants were provided with individual guidance on use of a smart phone application (MyFitnessPal, Inc., San Francisco, CA, USA) including completion details, and awareness of meal content, portion size, food weight and fluid intake. Individual records were checked daily by the same researcher to ensure satisfactory compliance. Diaries were assessed for daily mean caloric and macronutrient intake using Nutritics Professional Dietary Analysis software (Nutritics Ltd., Co., Dublin, Ireland) by the same researcher. General dietary consistency was reported between cohorts across the intervention period (see Table 4). In addition, as a means to quantify dietary HT intake (as the main polyphenol in OliP), individual food items were cross referenced against the U.S. Department of Agriculture (USDA) database on the flavonoid content of selected foods, and the Phenol-Explorer database. Estimated dietary HT intake (excluding supplementation) was consistent between groups and remained low demonstrating good overall compliance with the protocol requirement (see Section 3.2).

As a means to ensure satisfactory supplement compliance, participants were provided with a 16-day supply of allocated nutrition product and requested to complete a daily checklist when jars were consumed. As a secondary measure, all jars were returned on the final day of laboratory testing and cross-referenced with compliance records. Overall compliance was 98.1 ± 0.6% for PL and 99.0 ± 0.8% for OliP, demonstrating excellent adherence.

#### 2.3.4. Biochemical Assays

For all assays, plasma samples underwent a maximum of two freeze–thaw cycles and dilution tests were performed prior to sample testing for each kit to ensure the accuracy of assay conditions. All samples were randomized to avoid experimental bias.

*Malondialdehyde (MDA):* to determine plasma levels of MDA, the competitive ELISA kit (ab238537, Abcam plc, Cambridge, UK) was used. Manufacturer’s guidelines were followed but, in brief, plasma samples were incubated with MDA conjugate-coated wells of a 96-well plate for 10 min followed by addition of the anti-MDA antibody for 1 h at room temperature. Following wash steps, HRP-conjugated secondary antibody was incubated in the wells for 1 h and substrate solution was then added to develop a colorimetric output measured at 450 nm wavelength using a microplate reader (TECAN Sunrise^TM^, Mannedorf, Switzerland). A standard curve of MDA-BSA was prepared to extrapolate MDA adduct concentration (pmol·mL^−1^) from optical density (OD_450nm_) absorbance readings.

*Superoxide dismutase (SOD):* to assess SOD activity in plasma samples the colorimetric activity assay kit (ab65354, Abcam plc, Cambridge, UK) was utilized. Manufacturer’s guidelines were followed but, in brief, plasma was incubated with the WST dye and enzyme working solution for 20 min at 37 °C. The colorimetric output was measured at 450 nm wavelength using a microplate reader (TECAN Sunrise^TM^, Mannedorf, Switzerland). A standard curve of human SOD protein (ab112193) was used to extrapolate SOD activity as units per mL from optical density (OD_450nm_) absorbance readings.

*Catalase (CAT):* to measure CAT activity in plasma samples, the colorimetric activity assay kit (ab83464, Abcam plc, Cambridge, UK) was used. Manufacturer’s guidelines were followed but, in brief, plasma samples were incubated with hydrogen peroxide for 30 min at 25 °C prior to addition of the OxiRed Probe enriched developer solution for 10 min at 25 °C. The colorimetric output was measured at 570 nm wavelength using a microplate reader (TECAN Sunrise^TM^, Mannedorf, Switzerland). A standard curve of hydrogen peroxide was prepared to extrapolate CAT activity as nmol of hydrogen peroxide decomposed by CAT per minute per mL from optical density (OD_570nm_) absorbance readings.

*Reduced glutathione (GSH):* to determine levels of reduced GSH the fluorometric assay kit (ab138881, Abcam plc, Cambridge, UK) was used on plasma samples which had been deproteinized using the TCA sample preparation kit (ab204708). Manufacturer’s guidelines were followed but, in brief, deproteinized plasma samples were incubated with thiol green enriched assay buffer for 10–60 min at room temperature. A fluorometric plate reader (PerkinElmer VICTOR^®^, PerkinElmer LAS (UK) Ltd., Beaconsfield, Buckinghamshire, UK) was used to measure fluorescence at an excitation of 490 nm and emission of 520 nm wavelength. A standard curve of GSH was used to extrapolate GSH concentration (µM) from relative fluorescence units (RFU_490/520_).

HT: to determine whether the experimental product influenced plasma HT post-intervention compared with placebo, plasma HT was also determined in accordance with the method previously described (see Section 2.2.4).

### 2.4. Statistical Analysis

Statistical analyses were performed using SPSS (IBM, Version 26.0). Normality of data was verified by the Shapiro–Wilk test. Evaluation of outliers was undertaken using the interquartile range method. A mixed design repeated measures ANOVA was undertaken for main analyses (2 × 5 for bioavailability assessment; 2 × 2 × 4 for plasma biomarker assessment), with Bonferroni post hoc comparisons where applicable. Where sphericity was violated a Greenhouse–Geisser correction was applied. An alpha level of ≤0.05 was employed for statistical significance, with effect size (partial eta squared; η_p_^2^) also reported (small = 0.02, medium = 0.13, large = 0.26). Additionally, an unpaired t-test was employed for applicable data (e.g., participant characteristics, dietary and training load). Data are reported as the mean ± S.E.

## 3. Results

### 3.1. Bioavailability Assessment

**Figure 1:**
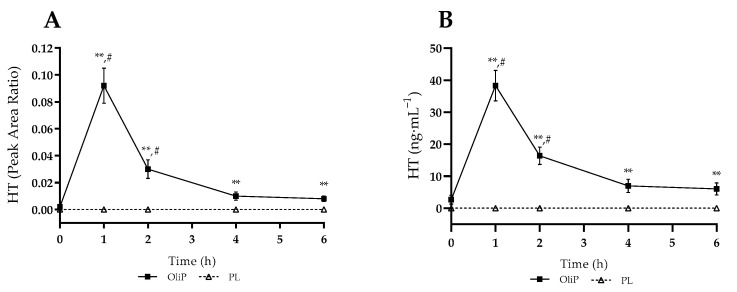
Plasma hydroxytyrosol (HT) responses following consumption of single serve of either Oliphenolia (OliP) or placebo (PL). (**A**) Instrument peak area ratio; (**B**) Converted HT concentration. ** denotes significant difference between conditions at timepoint (*p* ≤ 0.009). # denotes significant difference within OliP compared to baseline (*p* < 0.001).

Mean plasma HT measures are shown in Figure 1, expressed as instrument peak area ratio (PAR) and converted HT concentration. For PAR, there was a significant interaction effect (F = 25.60, *p* < 0.001, η_p_^2^ = 0.48), with all timepoints post-supplementation significantly different between conditions (*p* ≤ 0.009). Whilst no HT was detected within the PL trial, OliP significantly increased HT to 0.092 ± 0.013 (arbitrary units [AU]) at 1 h and remained elevated at 2 h compared to baseline (*p* < 0.001). A similar pattern was found for HT concentration, with a significant interaction effect reported (F = 21.07, *p* < 0.001, η_p_^2^ = 0.43). Peak plasma HT (C_max_) concentrations of 38.31 ± 4.76 ng·mL^−1^ occurred by 1 h (T_max_) and remained elevated until 4 h post supplementation (*p* < 0.001). OliP significantly elevated plasma HT concentration at all timepoints post-consumption compared with PL (*p* ≤ 0.003).

### 3.2. Main Intervention: Exercise Activity and Dietary Monitoring

Estimated mean exercise activity for each group across the main intervention period is shown in Table 3. No differences were reported for any of the variables (*p* > 0.05), indicating relative consistency in habitual training between groups. Likewise, mean dietary intake data across the main intervention are shown in Table 4. No significant differences were reported between groups for any of the reported variables (*p* > 0.05), with remaining minor energy intake (%) difference explained by contribution from habitual alcohol consumption. Estimation of dietary HT (excluding OliP supplementation) demonstrated a negligible mean intake of 9 ± 3 mg·d^−1^ for OliP compared with 12 ± 6 mg·d^−1^ for PL (*p* > 0.05). Mean body mass did not change within or between groups over the intervention (−0.27 ± 0.23 kg for OliP, 0.06 ± 0.25 kg for PL; *p* > 0.05).

### 3.3. Main Intervention Blood Analyses

#### 3.3.1. Plasma MDA

**Figure 2:**
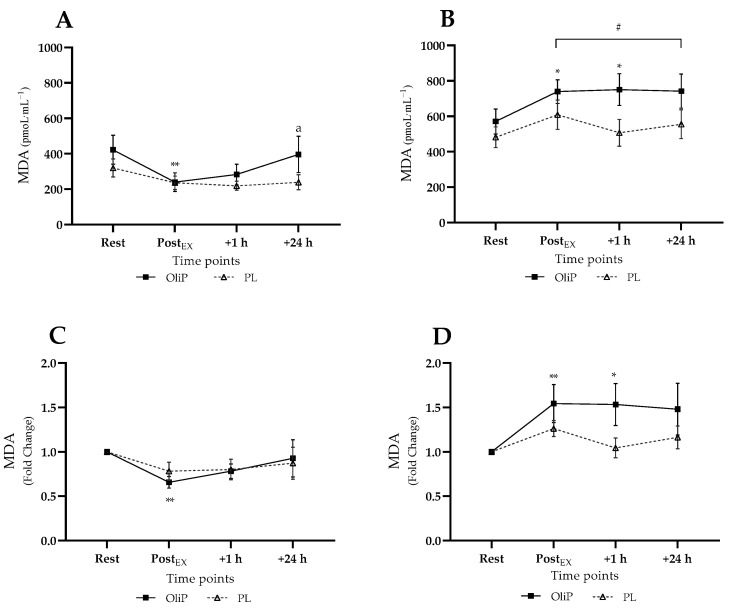
Plasma malondialdehyde (MDA) responses expressed in mean absolute (pmoL·mL^−1^) and fold change terms in participants that were supplemented with OliPhenolia^®^ (OliP) for 16 consecutive days or with a matched placebo (PL). (**A**,**C**) Pre-intervention, before supplementation; (**B**,**D**) Post 16-day supplement intervention. * denotes difference within OliP compared with resting levels (*p* ≤ 0.038); ** denotes difference within OliP compared to rest (*p* ≤ 0.008); a indicates difference within trial compared with PostEX (*p* = 0.05); # all significantly different to comparative timepoint pre-intervention within both PL and OliP (*p* ≤ 0.014).

Mean plasma MDA responses are shown in Figure 2, expressed in absolute and fold-change terms. A significant interaction effect was reported for absolute MDA concentration (F = 9.47, *p* < 0.001, η_p_^2^ = 0.26). Pre-intervention (tile A), absolute MDA levels did not increase post-exercise, with a significant reduction reported pre-post exercise only for OliP (*p* = 0.001). Post-intervention (tile B), for all time points post-exercise, MDA expression was significantly elevated compared with pre-intervention for both PL and OliP (*p* ≤ 0.005), indicating a relative increase in MDA in response to the exercise bout. However, post-intervention, absolute MDA only increased significantly within OliP immediately and 1 h post-exercise compared with resting levels (*p* = 0.032).

When expressed as fold change, a significant trial × time interaction effect was also found (F = 6.85, *p* < 0.001, η_p_^2^ = 0.20) pre-intervention, supporting the decrease in MDA noted for OliP post-exercise compared with rest (tile C, *p* = 0.002) and the converse increase in MDA for OliP only post-intervention (tile D, *p* ≤ 0.038). No differences were reported between conditions.

#### 3.3.2. Plasma SOD Activity

**Figure 3:**
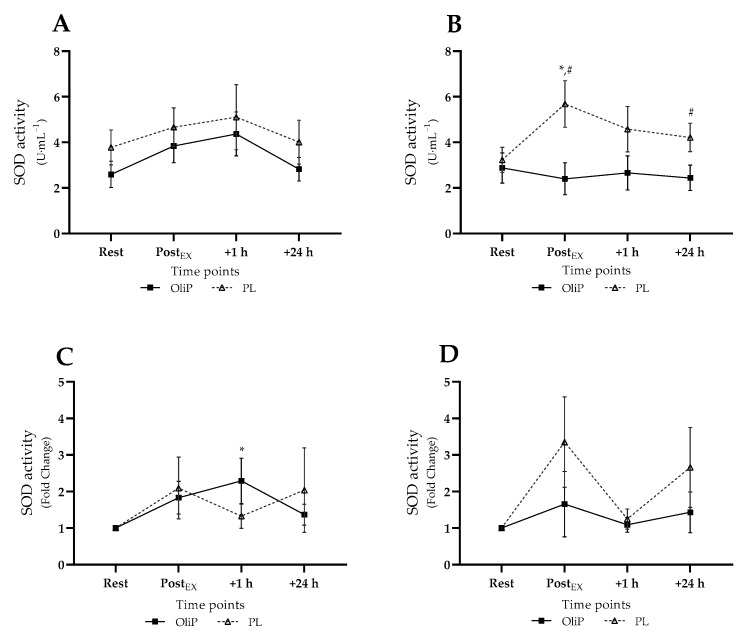
Plasma superoxide dismutase (SOD) activity expressed in mean absolute (U·mL^−1^) and fold change terms in participants that were supplemented with OliPhenolia^®^ (OliP) for 16 consecutive days or with a matched placebo (PL). (**A**,**C**) Pre-intervention, before supplementation; (**B**,**D**) Post 16-day supplement intervention. * denotes significant difference compared to rest within condition (*p* ≤ 0.038); # difference between conditions at timepoint (*p* ≤ 0.046).

Mean plasma SOD activity is shown in Figure 3, expressed in absolute and fold-change terms. A significant main effect was reported for time (F = 3.54, *p* = 0.018, η_p_^2^ = 0.12), and group (F = 4.31, *p* = 0.047, η_p_^2^ = 0.14). No differences were reported within or between groups pre-intervention. However, post-intervention, SOD activity significantly increased within PL from 3.23 ± 0.59 to 5.68 ± 1.06 U·mL^−1^ post-exercise (*p* = 0.038), with reported differences between conditions immediately (*p* = 0.016) and at 24 h post-exercise (*p* = 0.046, tile B). When expressed as fold-change, a significant interaction effect was reported (trial × time, F = 5.33, *p* = 0.004, η_p_^2^ = 0.17), with post hoc analysis highlighting only an increased SOD activity 1 h post-exercise compared with resting levels pre-intervention for OliP (*p* = 0.011; tile C). No other differences were reported.

#### 3.3.3. Plasma CAT Activity

**Figure 4:**
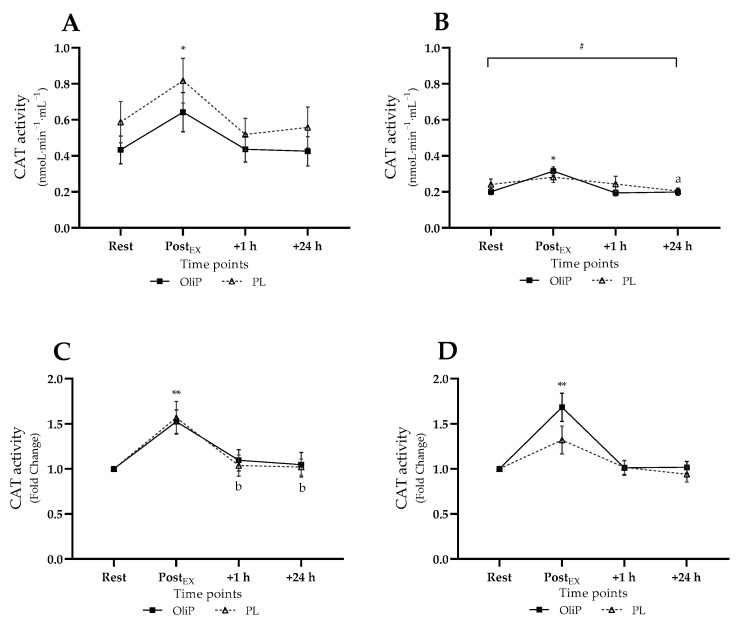
Plasma catalase (CAT) activity expressed in mean absolute (nmoL·min^−1^·mL^−1^) and fold change terms in participants that were supplemented with OliPhenolia^®^ (OliP) for 16 consecutive days or with a matched placebo (PL). (**A**,**C**) Pre-intervention, before supplementation; (**B**,**D**) Post 16-day supplement intervention. * denotes difference PostEx to all other timepoints within both OliP and PL pre-intervention, but only OliP post-intervention (*p* ≤ 0.04); a denotes difference compared with PostEx within PL only (*p* = 0.044); ** denotes difference within trial compared with rest (*p* ≤ 0.006), and all timepoints within OliP post-intervention (*p* ≤ 0.003); b denotes difference to PostEx within PL only (*p* ≤ 0.032); # all significantly different to comparative timepoint pre-intervention within both PL and OliP (*p* ≤ 0.024).

Mean plasma CAT activity is shown in Figure 4. A significant time × trial interaction effect was observed for absolute CAT activity (F = 4.56, *p* = 0.015, η_p_^2^ = 0.14). Pre-intervention, CAT activity increased post-exercise within PL and OliP compared to all other timepoints (*p* ≤ 0.04, tile A). However, post-intervention, all measures were reportedly lower to comparative pre-intervention timepoints for both PL and OliP (*p* ≤ 0.024, tile B). This highlighted a relative reduction in CAT activity in response to the exercise bout post-intervention. Post-intervention, CAT activity increased post-exercise within OliP compared to other timepoints (*p* ≤ 0.029).

When expressed as fold-change, a significant main effect was reported (F = 47.29, *p* < 0.001, η_p_^2^ = 0.64), demonstrating an increase in CAT activity in response to exercise pre-intervention (tile C) for both PL and OliP (*p* ≤ 0.006), with only PL demonstrating a significant reduction in CAT activity at 1 h and 24 h within condition (*p* ≤ 0.032). Post-intervention, CAT activity significantly increased post-exercise for OliP compared to all other timepoints within condition (*p* ≤ 0.003, tile D), whereas no change was observed for PL, further supporting absolute data.

#### 3.3.4. Plasma GSH

**Figure 5:**
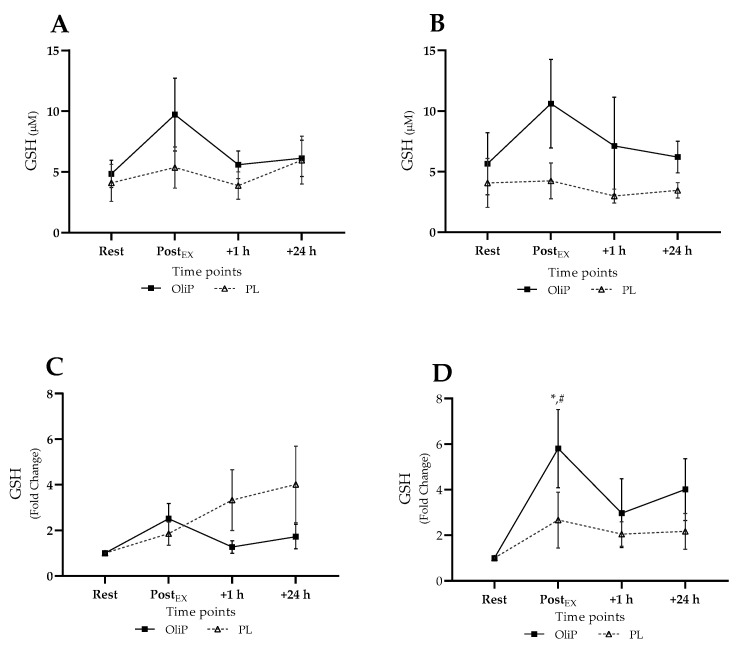
Plasma reduced glutathione (GSH) responses expressed in mean absolute (µM) and fold change terms in participants that were supplemented with OliPhenolia^®^ (OliP) for 16 consecutive days or with a matched placebo (PL). (**A**,**C**) Pre-intervention, before supplementation; (**B**,**D**) Post 16-day supplement intervention. * denotes significant difference compared to rest within trial (*p* = 0.05). # difference between trials at comparative timepoint for OliP only (*p* = 0.009).

Mean plasma GSH is shown in Figure 5, expressed in absolute and fold-change terms. A main effect for time was reported only (F = 2.94, *p* = 0.038, η_p_^2^ = 0.06), with no post hoc significance reported within conditions pre- or post-intervention. When expressed as fold-change, a significant trial × group interaction effect was reported (F = 4.10, *p* = 0.05, η_p_^2^ = 0.14), demonstrating a significant increase in GSH post-exercise, post-intervention (*p* = 0.05) and in comparison to PL (*p* = 0.009). No other differences were reported.

#### 3.3.5. Plasma HT

**Figure 6:**
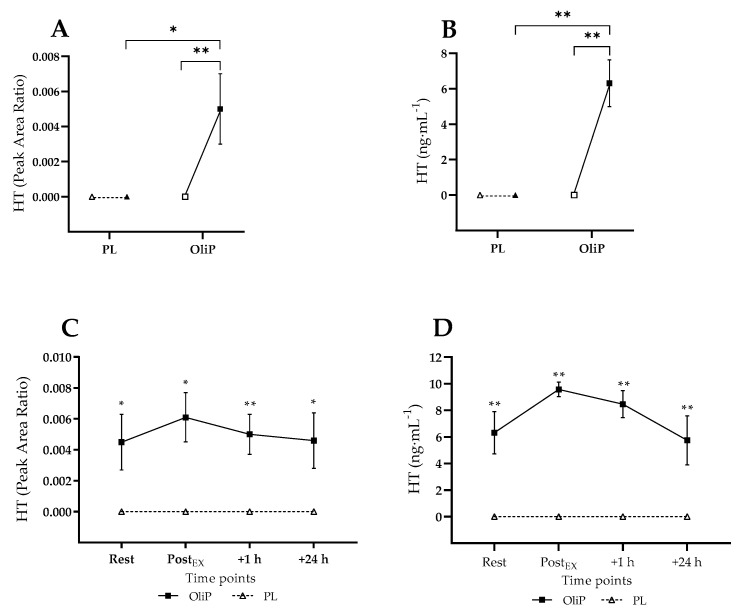
Plasma hydroxytyrosol (HT) responses expressed as instrument peak area ratio (**A**,**C**) and mean concentration (ng·mL^−1^; **B**,**D**) in participants that were supplemented with OliPhenolia^®^ (OliP) for 16 consecutive days or with a matched placebo (PL). (**A**,**B**) Resting data pre-intervention (open symbols) compared with post-intervention (filled symbols); (**C**,**D**) Responses post 16-day supplement intervention. Within (**A**,**B**) * indicates difference between comparative points (*p* = 0.029), whereas ** indicates highly significant difference between comparative points (*p* ≤ 0.003). Within (**C**,**D**) * indicates difference between conditions at timepoint (*p* ≤ 0.029), ** indicates stronger significance between conditions at timepoint (*p* ≤ 0.004).

Plasma HT concentrations at rest and in response to the exercise bout are shown in Figure 6. No HT was detected within either condition pre-intervention. However, OliP was significantly increased at rest following the intervention period when expressed as PAR (F = 5.61, *p* = 0.029, η_p_^2^ = 0.14) and converted concentration (F = 14.28, *p* = 0.001, η_p_^2^ = 0.43), in both cases being elevated in comparison to PL within trial (*p* ≤ 0.029) and compared to pre-intervention within condition (*p* ≤ 0.003, tile A and B). In response to the exercise bout, plasma HT was significantly elevated post-intervention at all timepoints (*p* ≤ 0.029) when expressed as PAR (main effect: F = 16.75, *p* < 0.001, η_p_^2^ = 0.50) and as HT concentration (main effect: F = 72.21, *p* < 0.001, η_p_^2^ = 0.81).

## 4. Discussion

### 4.1. Bioavailability Trial

The initial aim of this study was to investigate the bioavailability of HT as the main phenolic compound within commercially available OliP in healthy, adult volunteers, which, to our knowledge is the first study to do so. Importantly, our findings did not show evidence of HT recovery in plasma following consumption of the placebo supplement. This concurs with the independent analysis of the product (Table 1). In contrast, we found a significant 14-fold increase in plasma HT, with mean peak values of 38.3 ng·mL^−1^ (C_max_) occurring at 1 h (T_max_) following the consumption of a single serve of OliP, which remained elevated at 2 h compared with baseline levels. Whilst plasma HT remained significantly elevated across the 6 h trial for OliP compared with PL, HT concentrations were not significantly different to baseline by 4 h within the OliP trial. This infers that potential applications of OliP may coincide with this timing, and further supports company recommendations to consume two divided serves per day. However, future investigations should determine the fluctuation in plasma HT concentration when OliP is ingested twice per day (with ~6 h between serves), as recommended by the company.

Consumption of naturally derived HT is considered safe under the European Food Safety Authority (EFSA) at daily intakes of at least 200 mg HT·kg^−1^ BM for adults based on toxicity studies pertinent to establishing the no observed adverse effect level (NOAEL) [42]. Bioavailability of HT is largely influenced by first phase metabolism and intestinal transport [43], including microbiota oxidation and transformation [44], and supports findings elsewhere demonstrating peak HT concentrations between 0.5 and 1 h post-consumption in animal studies pending dose [43]. In human studies, consumption of a 99.5% pure HT supplement at a dose of 2.5 mg·kg^−1^ resulted in a HT C_max_ of 1.11 µmol·L^−1^ (range 0.47–2.23 µmol·L^−1^) with a T_max_ of 13 min (range 10–20 min) [45]. In the current study, we provided a fixed bolus of OliP which would have been broadly equivalent to a HT dose of ~0.4 mg·kg^−1^. Whilst these studies cannot be compared based on dose, both demonstrate a comparative increase in plasma HT concentrations associated to the provided dose of HT. However, differences in T_max_ may be partly explained by the lack of data preceding 1 h post-consumption, or that previous research [45] utilized a pure HT supplement delivered at a higher dose.

When 25 mL of extra virgin olive oil (EVOO) was administered to healthy participants [33], plasma C_max_ for HT and 3-O-methyl-hydroxytyrosol were captured at 32 and 53 min, respectively, with an estimated half-life of 2.4 h. Peak HT concentration was 25.8 µg·L^−1^, which was marginally lower than the current study. However, this may be associated with the concentration or relative potency of HT in OliP (~29 mg per serve) along with phenolic derivatives including HT glucoside (~8 mg per serve) and oleuropein aglycone (~9 mg per serve). Additionally, the contribution of other phenols including oleuropein (which can be converted to HT in the gut via hydrolysis [46]) as part of concentrated OliP may, in part, explain a higher C_max_ with OliP in comparison to previous research investigating EVOO [33]. As OliP is a naturally derived and concentrated olive fruit water phytocomplex, it is also feasible that other phenolic derivates may act as potent antioxidants within the gut, potentially preserving HT (and related compounds), leading to a sustained absorption curve compared with EVOO or pure HT-only compounds.

It should be noted that Domínguez-Perles et al. [43] also demonstrated that increasing dose of HT ingestion does not follow a linear pharmacokinetic pattern, potentially suggesting a saturation threshold. In the current study, an acute bolus of OliP was consumed and it is unknown whether a greater C_max_ would have occurred with higher doses or whether absorption curves can be sustained with more regular ingestion. Future research should therefore investigate the impact of OliP dose and timing on plasma HT concentrations, as well as identifying whether T_max_ occurs sooner than 1 h post ingestion. Additionally, specific analysis of other compounds associated with HT contribution from OliP including HT glycoside, tyrosol, oleuropein and ligstroside (especially in their conjugated forms), and related metabolites, including homovanillic acid, 3-O-methyl-hydroxytyrosol, and 3,4-dihydroxyphenylacetic acid (DHPA), would also support understanding of the efficacy of OliP.

### 4.2. Main Intervention

The second aim of this study was to assess the impact of 16 days’ supplementation of OliP on selected antioxidant enzymes and markers of oxidative stress in response to aerobic exercise. Prior to supplementation, no differences were reported between groups in response to exercise. Both SOD and CAT activity appeared to follow the expected trend pre-post exercise, demonstrating a modest increase in oxidative stress, although recovering within 24 h post exercise. High inter-individual variability was reported for GSH, likely explaining the lack of significance observed for absolute or fold-change data pre-intervention. Surprisingly, MDA did not increase post-exercise as expected, and in fact was significantly suppressed immediately post-exercise within OliP only, recovering by 1 h post exercise.

Previous studies have found either no effect or a modest effect on plasma MDA levels following shorter duration (30 min at 70–80% $\dot{V}$O_2_max; [47,48,49]) or maximal exercise [50]. In contrast, others have highlighted the relative importance of high intensity bouts [38] in eliciting a ‘stress’ response. Steady-state moderate intensity exercise (60–70% $\dot{V}$O_2_max) for 30–60 min followed by hard domain (90% $\dot{V}$O_2_max; [51]) or performance efforts [52] have provoked greater MDA responses. In the current study, we therefore opted to employ a sustained but demanding exercise bout (50 min at ~75% $\dot{V}$O_2_max, ~90%HRmax) followed by hard domain intermittent efforts to complete a 1 h bout. Pre-intervention, it is therefore unclear why we did not observe an increase in MDA post-exercise, considering the observed expected changes in SOD and CAT activity. Whilst mixed findings have been previously reported for exercise-related changes in MDA [53], as participants were requested to fully rest prior to the exercise bout, it is possible this influenced MDA observations specific to the pre-intervention laboratory test only (based on elevated findings following repetitive, habitual exercise training 16 days later). Alternatively, inclusion of other markers of oxidative stress (e.g., F_2_-isoprostanes or protein carbonyls) may have yielded clearer insights [53].

An important finding from the current study was that after 16 days of supplementation with OliP, plasma HT was increased at rest (~6.3 ng·mL^−1^) and following exercise (peak ~ 9.6 ng·mL^−1^) compared with PL and pre-intervention measures. As the main component within OliP is naturally derived HT and oleuropein-aglycone di-aldehyde (3-4-DHPEA-EDA), this demonstrates that short-term use of OliP increased systemic HT concentrations, either in response to the final bolus or accumulation over the 16-day intervention. Previous research has demonstrated very low levels of plasma HT (~0–0.2 ng·mL^−1^ [32]) increasing to ~4 ng·mL^−1^ [33,54] following acute ingestion of EVOO. The current study demonstrates that OliP may have important benefits in increasing HT from natural sources, and in itself warrants further investigation.

A second key finding was that OliP appeared to have modest antioxidant effects, which supports previous research utilizing olive mill wastewater [55]. Compared with PL, OliP suppressed SOD activity, immediately post-exercise and in the proceeding 24 h. By converting O_2_^•−^ to H_2_O_2_ and O_2_, a reduction in SOD activity potentially indicates a lower oxidative stress response. HT has been associated with a direct scavenging effect on O_2_^•−^ in vitro [56], although in vivo evidence has been contested [57]. This ROS scavenging effect of HT may also extend to H_2_O_2_ [57,58], and hence lower production of ^•^OH, thereby limiting acute mitochondrial or DNA damage. This is particularly relevant considering habitual exercise and dietary patterns were maintained across the intervention. In the current study, we utilized aerobic exercise as an indirect model of stress; however, this finding may have important clinical relevance where reduced oxidative stress may be pertinent (e.g., cardiovascular disease, arthritis, neurodegenerative disorders and cancer [59,60,61]). Furthermore, as polyphenols are established to impact anti-inflammatory pathways through MAPK and NF-kB signaling, and related antioxidant enzyme pathways [62,63], OliP may have adjunct therapeutic applications.

Post-intervention, GSH was elevated post-exercise with OliP (when expressed as fold-change), further supporting transient reductions in oxidative stress compared with PL. This may, in part, be explained via a reduction in H_2_O_2_ with OliP, based on changes in SOD activity. As GSH plays an essential role in maintaining redox balance, this could be pertinent to acute recovery [64]. Interestingly, CAT activity was suppressed post-intervention for both conditions, and whilst no differences were reported between OliP and PL, within group it was noted that CAT activity was only elevated post-exercise with OliP (especially when expressed as fold-change). Whilst this could indicate a subtle pro-oxidant effect, absolute levels suggest this is unlikely.

Although our findings infer that OliP may have direct ROS scavenging effects based on HT (and related phenolic compounds), the relatively low plasma concentrations found (<40 ng.mL^−1^) likely indicate this may not be the case [65]. Indeed, there is evidence that regular consumption of polyphenol compounds (including HT) may impact the Nrf2 and subsequent antioxidant response element (ARE) pathways, thereby supporting endogenous antioxidant mechanisms [65,66,67]. Alternatively, phenolic compounds within OliP may be inhibiting NADPH oxidase, thereby reducing O_2_^•−^ production, and associated SOD requirements. Further research is warranted to investigate the specific antioxidant mechanisms in relation to OliP based on current findings.

From an exercise perspective, there is current debate as to the efficacy of antioxidants to support training mechanisms. A reduction in oxidative stress may be pertinent for calcium handling, contractile force development and O_2_kinetics, impacting exercise workload and/or subsequent performance, as well as post-exercise recovery [56]. In contrast, from a hormetic perspective, an acute prooxidant effect from exercise may support upregulation of endogenous antioxidant enzymes, and specific use of antioxidant nutrients may conflict with such mechanisms [68,69,70,71]. Other studies utilizing various polyphenol supplementation have noted conflicting results, either showing limited impact [72], a reduction in oxidative stress markers [73,74,75,76] or increased lipid peroxidation [77]. This highlights the complexity, and both the negative and positive effects of oxidative stress in maintaining homeostasis following any form of stressor. However, based on the current findings, it is proposed that OliP may result in an indirect reduction in O_2_^•−^ acutely, or via Nrf2/ARE signaling, which may support oxidative metabolism during exercise (i.e., lower O_2_cost) by limiting ROS associated mitochondrial damage. From this standpoint, there may be a place for OliP in supporting recreationally active individuals undergoing intensive and/or repetitive demanding exercise; however, further training specific studies are needed to confirm this.

In contrast to pre-intervention levels, our controlled exercise resulted in a significant increase in MDA for both conditions (PL and OliP) post-exercise, for up to 24 h. It is feasible that the accumulated training load (whilst non-significant between conditions) over the intervention period may have contributed to this, despite participants resting prior to the laboratory visit. Whilst no differences were reported between conditions, it is noteworthy that MDA was significantly elevated immediately following exercise and after 1 h recovery for OliP only. Although considered low, this could indicate a mild pro-oxidant effect of OliP, which may align with hormetic theories associated with positive training adaptations [78]. However, based on absolute MDA levels it is proposed this is unlikely, and further research is warranted to confirm these findings, particularly following repeated exercise (considering the current data represent an acute setting) or longer-term training protocols.

### 4.3. Study Limitations and Future Directions

It is important to recognize several limitations of the current study. Whilst selected antioxidant enzymes and markers of oxidative stress were based on previous research, inclusion of other biomarkers of lipid and protein oxidation (e.g., F_2_-isoprostanes, protein carbonyls) and DNA damage (e.g., 8-hydroxy-2-deoxyguanosine (8-OHdG)) would have provided further insights. Additionally, we measured reduced GSH to indirectly assess oxidative stress; however, inclusion of oxidized GSH (GSH:GSSH) or GPx would have supported mechanistic actions of OliP. Whilst we ascertained the impact of OliP on plasma HT levels following a 16-day intervention, additional measures for antioxidant capacity, as well as Nrf2/ARE signaling pathways via biopsy quantification, would also have been beneficial. It is also unknown whether other phenolic compounds in OliP were having indirect or direct antioxidant effects, and hence based on these findings further research is warranted to explore contributing compounds from this olive-derived phytocomplex.

Another recognized limitation is that whilst we utilized a single batch of OliP, research on inter-batch variance should be investigated. Previous research has noted considerable batch variability with other polyphenol products [79], potentially explaining differences between study findings. Adding to this, the complexity of polyphenol metabolism is exacerbated by both the metabolic fate of conjugated and unconjugated phenolic compounds, as well as influence from inter-individual microbiome variability. Therefore, individual responses may equally be variable based on polyphenol metabolism, as well as interplay from other lifestyle factors (e.g., diet, circadian stress and sleep quality).

Whilst this study demonstrates that OliP may have modest antioxidant effects, habitual endogenous antioxidant patterns were only measured in response to acute exercise in a recreationally active cohort. Further research is warranted to ascertain whether OliP has differing effects based on gender, training status or the type/intensity of habitual exercise undertaken. It would also be meaningful to explore both the dose and time course effect (>16-days) of OliP in relation to sustained training (e.g., marathon running), especially in light of animal studies demonstrating a prooxidant performance enhancing effect at high doses [80]. In addition, based on suppression of SOD in response to an acute exercise stress, assessment of OliP on inflammatory markers is also warranted pertinent to exercise-induced muscle damage and clinical states when functional movement may be impacted (e.g., arthritis, fibromyalgia).

## 5. Conclusions

This is the first study to investigate the use of a natural olive-derived phytocomplex (OliPhenolia^®^) in an exercise domain. Findings demonstrated that HT bioavailability from OliP peaked at 1 h, and whilst remaining elevated across the assessed period compared with PL, was non-significant compared to baseline within condition by 4 h. A 16-day supplementation period of OliP suppressed SOD activity and increased GSH in the 24 h after a 60 min demanding aerobic exercise bout, demonstrating a modest antioxidant effect in healthy adults. These findings may support commercial applications, and may be beneficial in an acute exercise setting. However, further research is required to ascertain the longer term and dose response effects of OliP in relation to repeated exercise or sustained training periods, as well as clinical domains pertinent to reduced oxidative stress.

## Figures and Tables

**Table 1:** Independent analysis of OliPhenolia^®^ (OliP) and placebo (PL) products.

	OliP (mg·L^−1^)	PL (mg·L^−1^)	OliP (/28 mL Serve)	PL (/28 mL Serve)
Total phenolic profile	11,282	1748	315.9	48.9
Hydroxytyrosol (HT)	1030	0	28.8	0
3-4-DHPEA-EDA	810	0	22.7	0
Verbascoid	570	0	16.0	0
Oleuropein aglycone	310	0	8.7	0
Hydroxytyrosol glucoside	290	0	8.1	0
Tyrosol glycosylated derivatives	240	0	6.7	0
Hydroxy-verbascoid isomer 2	220	0	6.2	0
Hydroxy-verbascoid isomer 1	200	0	5.6	0
*p*-coumaroyl secologanoside	190	0	5.3	0
Isoverbascoid	0	0	0	0
Rutin	0	0	0	0
Chlorogenic acid	0	0	0	0
Caffeic acid	0	0	0	0
Luteolin-7-O-Glucoside	0	0	0	0
Nuzhenide	0	0	0	0
*p*-HPEA-EDA	0	0	0	0
Caffeoyl secologanoside	0	0	0	0

Analytical Group SRL, Florence, Italy; 3-4-DHPEA-EDA = Oleuropein-aglycone di-aldehyde; *p*-HPEA-EDA = Decarboxymethyl ligstroside aglycone.

**Table 2:** Baseline participant characteristics, including intervention group distribution for those supplemented with OliPhenolia^®^ (OliP) for 16 consecutive days or with a matched placebo (PL).

	Overall	OliP	PL
	(*n* = 29; 20 M, 9 F)	(*n* = 15; 11 M, 4 F)	(*n* = 14; 9 M, 5 F)
Age (years)	42 ± 2	42 ± 3	42 ± 3
Height (m)	1.76 ± 0.02	1.77 ± 0.03	1.75 ± 0.03
Body mass (kg)	71.08 ± 2.14	73.57 ± 2.44	68.41 ± 3.52
Fat free mass (kg)	57.67 ± 2.31	59.33 ± 3.05	55.89 ± 3.56
Body mass index (kg·m^2^)	22.9 ± 0.4	23.5 ± 0.4	22.3 ± 0.7
Body fat (%)	18.7 ± 1.8	19.5 ± 2.2	17.8 ± 3.0
$\dot{V}$O_2max_ (L·min^−1^)	3.53 ± 0.16	3.56 ± 0.22	3.49 ± 0.24
$\dot{V}$O_2max_ (mL·kg^−1^·min^−1^)	49.55 ± 1.67	48.25 ± 2.50	50.95 ± 2.22

Data presented as mean ± S.E. M = male; F = female. No significant differences reported between groups.

**Table 3:** Estimated exercise activity and training load parameters across the 16-day intervention period for those consuming OliPhenolia^®^ (OliP) or a matched placebo (PL).

	OliP	PL
Average session duration (mins)	59 ± 4	67 ± 5
Average session heart rate (b·min^−1^)	135 ± 4	140 ± 4
Average session perceived exertion (0–10)	5.2 ± 0.3	4.8 ± 0.3
Mean daily training load (AU)	240 ± 35	289 ± 36
Accrued intervention training load (AU)	3832 ± 552	4617 ± 574
Estimated training monotony (AU)	1.07 ± 0.06	1.45 ± 0.21
Estimated training strain (AU)	4371 ± 829	5959 ± 1197

No significant differences reported between groups (*p* > 0.05). Session perceived exertion based on 0–10 visual analogue scale.

**Table 4:** Mean dietary intake across the 16-day intervention for those consuming OliPhenolia^®^ (OliP) or a matched placebo (PL).

		OliP	PL
Energy Intake	(kcal·d^−1^)	2233.8 ± 155.5	2397.8 ± 143.7
	(kcal·kg^−1^·d^−1^)	30.3 ± 2.2	35.9 ± 2.3
Carbohydrate	(%EI)	44.2 ± 1.6	44.4 ± 1.8
	(g·d^−1^)	246.4 ± 19.7	270.7 ± 23.0
	(g·kg^−1^·d^−1^)	3.3 ± 0.3	4.0 ± 0.3
Protein	(%EI)	18.6 ± 07	18.1 ± 0.9
	(g·d^−1^)	104.7 ± 9.3	108.1 ± 8.5
	(g·kg^−1^·d^−1^)	1.4 ± 0.1	1.6 ± 0.1
Fat	(%EI)	35.5 ± 1.2	36.3 ± 1.8
	(g·d^−1^)	88.0 ± 6.3	94.8 ± 5.1
	(g·kg^−1^·d^−1^)	1.2 ± 0.1	1.4 ± 0.1

No significant differences reported between groups (*p* > 0.05).

## Data Availability

The data presented in this study are available on request from the corresponding author. The data are not publicly available due to ethical considerations, in accordance with consent provided by participants on the use of confidential data.
